# The Basic Research of the Combinatorial Therapy of ABT-199 and Homoharringtonine on Acute Myeloid Leukemia

**DOI:** 10.3389/fonc.2021.692497

**Published:** 2021-07-14

**Authors:** Yuanfei Shi, Jing Ye, Ying Yang, Yanchun Zhao, Huafei Shen, Xiujin Ye, Wanzhuo Xie

**Affiliations:** ^1^ Department of Hematology, The First Affiliated Hospital, College of Medicine, Zhejiang University, Hangzhou, China; ^2^ Sports Medicine Department, Beijing Key Laboratory of Sports Injuries, Peking University Third Hospital, Beijing, China; ^3^ Institute of Sports Medicine, Peking University, Beijing, China; ^4^ Department of Gynaecology and Obstetrics, Northwest Women’s and Children’s Hospital, Xi’an, China

**Keywords:** ABT-199, homoharringtonine, cancer, acute myeloid leukemia, combinatorial therapy, molecular mechanisms, basic research

## Abstract

**Background:**

Existing research shows that ABT-199, as a first-line drug, have been widely used in hematological malignancies, especially in leukemia, but the clinical efficacy of single drug therapy was limited part of the reason was that BCL-2 inhibitors failure to target other anti-apoptotic BCL-2 family proteins, such as MCL-1. In this case, combination therapy may be a promising way to overcome this obstacle. Here, we investigate the preclinical efficacy of a new strategy combining ABT-199 with homoharringtonine (HHT), a selective inhibitor of MCL-1 may be a promising approach for AML treatment as these two molecules are important in apoptosis.

**Methods:**

A Cell Counting Kit-8 (CCK8) assay and flow cytometry were used to determine the half-maximal inhibitory concentration (IC50) value and cell apoptosis rate, respectively. The flow cytometry results showed that combined treatment with HHT and ABT-199 caused apoptosis in AML patient samples (n=5) but had no effect on normal healthy donor samples (n=11). Furthermore, we used a Western blot assay to explore the mechanism underlying the efficacy of HHT combined with ABT-199. Finally, antileukemic activity was further evaluated *in vivo* xenograft model.

**Results:**

Our results indicated that ABT-199 combined with HHT significantly inhibited cell growth and promoted apoptosis in both AML cell lines and primary AML tumors in a dose- and time-dependent manner. Moreover, HHT combined with ABT-199 suppressed AML cell growth and progression *in vivo* xenograft model.

**Conclusions:**

Our research found that HHT combined with ABT-199 exerted its anti-leukemia effect by inducing apoptosis through the treatment of AML *in vitro* and *in vivo*.

## Introduction

Acute myeloid leukemia (AML) is a common and severe type of acute leukemia, especially in adults. In the United States, nearly 20,000 patients suffer from AML every year. Worse still, AML causes over 10,000 deaths per year. The 5-year survival rates, which are 65% for children and 26% for adults, remain quite low (1, 2). It has been reported that apoptosis evasion is associated with tumorigenesis and drug resistance (3). The upregulation of antiapoptotic BCL-2 family members and MCL-1 functions are two typical approaches exploited by cancer cells to escape apoptosis (4).

Targeted therapy has emerged as a promising treatment strategy for AML, which is resistant to chemotherapy. BCL-2 plays an important role in chemoresistance as an effective antiapoptotic protein (5, 6). Targeting BCL-2 with BH3 mimetics, such as ABT-199 (venetoclax), shows superior effects on lymphoma, especially when combined with homoharringtonine (HHT) (7).

ABT-199, a selective inhibitor of BCL-2, shows remarkable efficacy in a large number of cancers (6, 8). The emergence of ABT-199 provides an opportunity to study the function of BCL-2 inhibition. Other studies have shown that targeting BCL-2 *via* ABT-199 can effectively induce apoptosis in AML. Furthermore, overexpression of MCL-1, another antiapoptotic protein, renders leukemia cells resistant to both ABT and its predecessor ABT-737 (9, 10).

HHT, an omacetaxine mepesuccinate, has been widely studied and used in China as a classic antileukemic drug. However, the precise targets of HHT remain unclear (11, 12). Our findings indicated that HHT could potentiate the cytotoxicity of ABT-199 to leukemia cells. Moreover, a regimen combining ABT-199 with HHT was highly active against primary cells obtained from patients with refractory or relapsed AML.

## Materials and Methods

### Chemicals and Reagents

ABT-199 was purchased from Selleck Chemicals (Houston, TX, USA). HHT (Zhejiang Minsheng Pharmaceutical, Zhejiang, China) was dissolved in sterile phosphate-buffered saline (PBS) at 1 mg/mL and stored at -20°C. HHT was diluted to the required concentrations in subsequent experiments with culture medium.

### Cell Culture

AML cell lines (OCL-AML2, OCL-AML3, MOLM-13, and MV4-11) were purchased from the American Type Culture Collection (ATCC, Manassas, VA, USA). Cells were cultured in a humidified incubator at 37°C and 5% CO_2_ in RPMI 1640 medium (HyClone, Logan, UT, USA) containing 10% fetal bovine serum (FBS) (Gemini, Sacramento, CA, USA).

### Patient Samples

Primary AML cells were extracted from patients newly diagnosed with AML at the Department of Hematology, the First Affiliated Hospital of Zhejiang University. Bone marrow samples were collected from healthy hematopoietic stem cell transplantation donors (n=11). The characteristics of the patients with AML are shown in **Table 3**. The experiment was conducted by the guidelines of the Declaration of Helsinki and was approved by the Ethics Committee of the Faculty of the First Affiliated Hospital of Zhejiang University. IRB approval information is shown in **Supplemental 4**.

### Cell Viability Assay

The cytotoxic effects of ABT-199 and HHT on AML cell lines were determined by a Cell Counting Kit-8 (CCK8; Dojindo, Kumamoto, Japan) assay. Cells (2×10 (4) cells/well) were seeded in 96-well plates containing 100 µL growth medium and treated with designated doses of ABT-199 or HHT alone or in combination at 37°C in a humidified 5% CO_2_-95% air incubator for 24 h or 48 h; the optical densities (O.D.s) at the dual wavelengths of 450/630 nm were determined using a microplate reader (BIO-TEK EPOCH, USA).

### *In Vitro* Clonogenicity Assay

To evaluate colony-forming abilities following drug treatment, OCL-AML2, OCL-AML3, and MOLM-13 cells (2×10^5^/well) in the logarithmic growth phase were seeded in 24-well plates and then treated with 80 nM ABT-199 or 16 nM HHT alone or both molecules. After 24 h, the cells were washed and further cultured in complete methylcellulose medium at a cell density of 500 cells/well in 3.5-cm dishes for 14 days. Colonies consisting of at least 50 cells were counted and analyzed for clonogenicity.

### Apoptosis Assay

To assess apoptosis, OCL-AML2, OCL-AML3, MOLM-13, and MV4-11 cells were cultured and treated with different doses of ABT-199 or HHT alone or in combination for 24 h or 48 h and then double-labeled with Annexin-V-FITC/PI (eBioscience, San Diego, California, USA) for 20 min at room temperature in the dark according to the manufacturer’s instructions. The stained cells were analyzed with a NovoCyte flow cytometer (ACEA Biosciences, Inc.) with NovoExpress software. Apoptotic cells were defined as Annexin-V positive.

### Western Blot Analysis

Cell lysates were prepared using RIPA protein lysis buffer (Beyotime, Nantong, China). MOLM-13 cells (2×10^5/^mL) were cultured with 5 nM ABT-199 or 4 nM HHT alone or the two drugs in combination for 24 h because no obvious apoptosis was observed at this time. The specific antibodies used in this study included those specific for β-actin, MCL-1, Caspase-3, BCL-2, FLT3, and (rabbit monoclonal antibodies, 1:1000, Cell Signaling Technology). Proteins were detected by the addition of horseradish peroxidase (HRP)-conjugated secondary antibody. Signals were detected using the ECL Western Blotting Detection Kit (Gene-Flow, Staffordshire, UK).

### Xenograft Tumor Model

Twenty-four female NOD/SCID mice (4-6 weeks of age, nonpregnant, female, and 16-18 g) were purchased from the Nanjing Biomedical Research Institute of Nanjing University (Nanjing, China). OCL-AML3 cells (5×10^6^) were subcutaneously injected into the front-left region of the NOD/SCID mice. When tumor volumes reached approximately 75 mm^3^, the mice were randomly divided into four groups: the vehicle, ABT-199, HHT, and combination groups (n=5). The mice were treated with the vehicle (the same volume of normal saline), ABT-199 (50 mg/kg/day), HHT (1.0 mg/kg/day), or the corresponding doses of ABT-199 and HHT by oral gavage for 2 successive weeks. Tumor volume (V) was determined by the equation V = (L × W^2^)/2, where L is tumor length and W is tumor width.

### Statistical Analysis

Statistical analyses were conducted using Prism software v6.0 (GraphPad Software, La Jolla, CA, USA); at least three independent experiments were performed and compared using Student’s t-test. Multigroup comparisons were performed using one-way analysis of variance (ANOVA) followed by the Bonferroni *post hoc* test. Survival was estimated using Kaplan-Meier analysis and compared using the log-rank test. We used Calcusyn v2.0 software to calculate the “combination index” (CI) of the drug combination treatment to describe synergism (CI < 1), addictive effect (CI = 1), or antagonism (CI > 1) (13). P values < 0.05 were considered statistically significant. Statistical analyses were performed using SPSS 20.0 software (La Jolla, CA).

## Results

### Combination of ABT-199 and HHT Exerted Antileukemic Activity in Diverse AML Cell Lines

**Figure 1:**
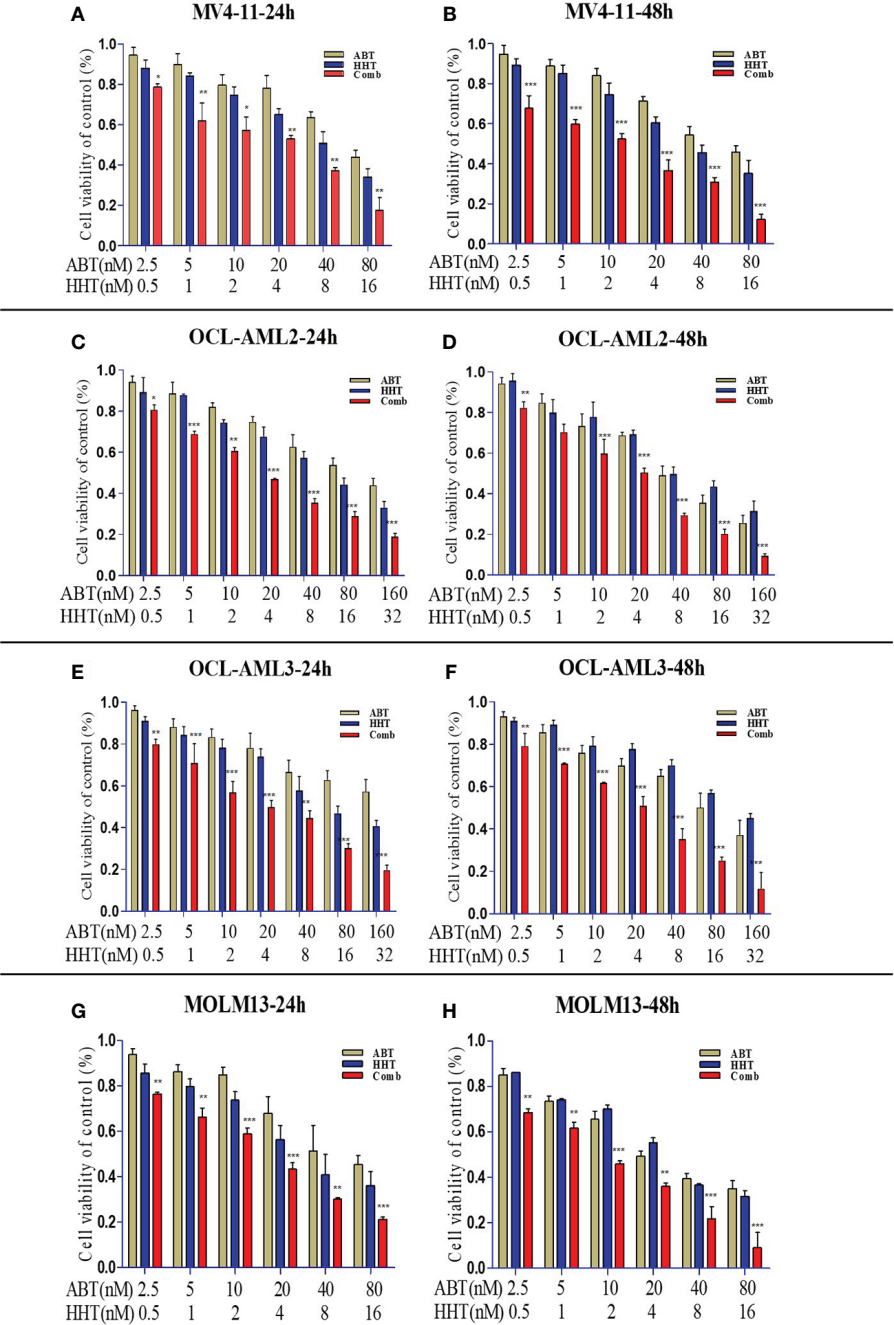
AML cell lines (OCL-AML2, OCL-AML3, MOLM13, and MV4-11) were treated with various doses of ABT-199 or HHT alone or in combination for 24 h or 48 h **(A–H)**. The percent viability is normalized to the percent viability of the DMSO-treated control. Values are expressed as the mean ± S.D. @ of three independent experiments (*P < 0.05, **P < 0.01, and ***P < 0.001).

First, we used an MTT assay to examine the viability of AML cell lines treated with ABT-199 or HHT alone or in combination. The concentrations of ABT-199 and HHT are shown in **Figures 1A–H**. We found that AML cell lines treated with both ABT-199 and HHT showed a much better inhibitory effect especially in OCL-AML2, OCL-AML3, MOLM-13, and MV4-11cell lines with FLT3-ITD mutation than those treated with each reagent alone in a time-dependent manner. The half-maximal inhibitory concentration (IC50) values (**Table 1**) of ABT-199 and HHT were lower at 48 h than at 24 h in all cell lines (P < 0.001). Percent viabilities of the DMSO-treated control of the ABT-199 and HHT in the ratios (3:1 and 7:1) were shown in the **Supplemental Figures 1**, **2**.

**Table 1:** IC_50_ values of ABT-199 and HHT as single agent in AML cells.

AML cell lines	IC_50_ at 24h(nM)		IC_50_ at 48h(nM)	
	ABT-199	HHT	ABT-199	HHT
MV4-11	12.54 ± 2.69	10.15 ± 1.82	9.07 ± 1.30	5.32 ± 1.21
MOLM13	9.81 ± 1.13	6.060 ± 0.84	2.86 ± 1.51	1.54 ± 0.89
OCL-AML2	126.2 ± 2.43	65.00 ± 1.96	15.67 ± 0.65	4.67 ± 0.79
OCL-AML3	34.1 ± 0.49	23.94 ± 0.53	3.87 ± 0.69	4.85 ± 0.83

IC50: Half maximal inhibitory concentration.

### Combination Treatment With ABT-199 and HHT Synergistically Induced Apoptosis in Both AML Cell Lines and Primary AML Samples

**Figure 2:**
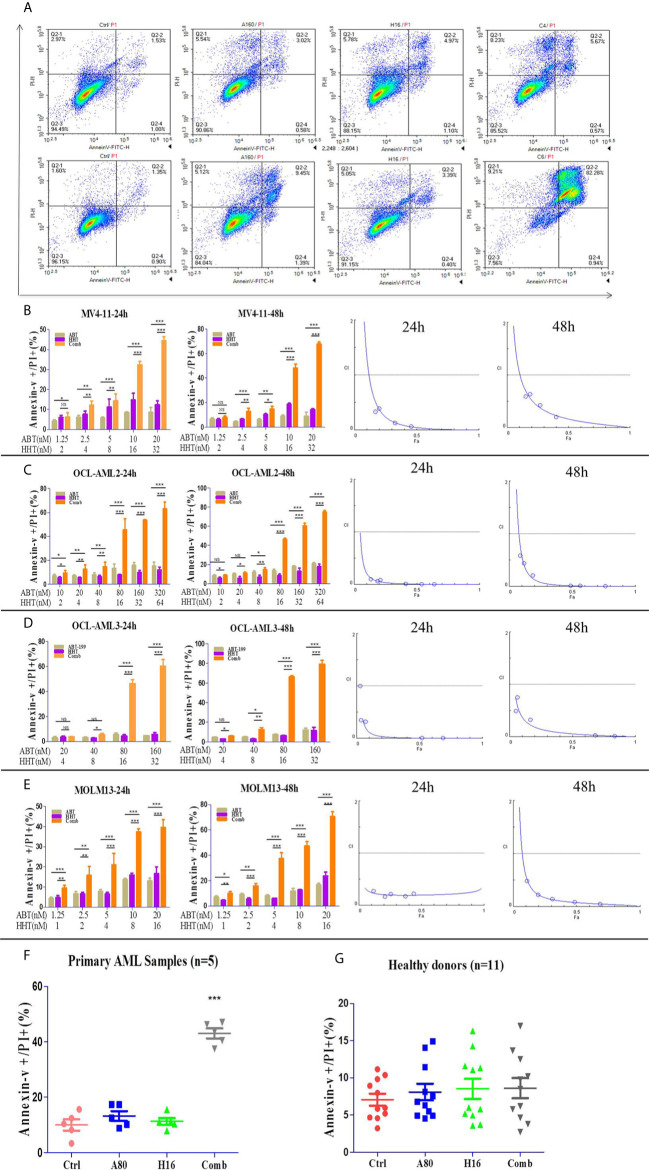
The percentage of apoptotic cells was examined with a NovoCyte flow cytometer. ABT-199 combined with HHT resulted in significant increases in the apoptosis rate in AML cell lines **(A)**. The same results were observed in primary leukemia samples **(B–F)**. The combination regimen exhibited minimal toxicity to normal peripheral blood mononuclear cells obtained from healthy donors **(G)** (*P < 0.05, **P < 0.01, and ***P < 0.001; NS, not significant).

We investigated the effects of ABT-199 and HHT alone or in combination on AML cell lines. OCL-AML2, OCL-AML3, MOLM13, and MV4-11 cells were exposed to the indicated concentrations of ABT-199 with or without HHT for 24 h or 48 h. As shown in **Figure 2A**, ABT-199 or HHT alone was unable to induce apoptosis, while the combination could significantly increase apoptosis in all of the tested AML cell lines. Combination index (CI) values were calculated according to the median effect method of Chou and Talala **(** **Table 2** **)**. A CI value of less than 1.0 indicates a synergistic effect. Then, we further confirmed the antileukemic activity of ABT-199 combined with HHT in primary samples (n=5). The clinical characteristics of the donor AML patients are summarized in **Table 3**. Consistent with the antileukemic activity of ABT-199 plus HHT observed in the AML cell lines **(** **Figures 2B–E** **)**, exposure of primary AML cells to ABT-199 and HHT resulted in remarkable apoptosis **(** **Figure 2F** **)**. In contrast, the combination of ABT-199 and HHT displayed minimal toxicity to normal peripheral blood mononuclear cells obtained from healthy donors (n=11) **(** **Figure 2G** **)**. The CI value of other ratios of ABT-199 and HHT were shown in the **Supplemental Figure 3** **(3:1)** and **Supplemental Figure 4** **(7:1)**. The CI values are listed in **Supplemental Table 1**. These findings indicated that the combination of ABT-199 and HHT might be a promising therapy for AML that spares normal hematopoietic cells.

**Table 2:** The effect of synergistic inhibition in AML cell lines.

MV4-11					
Concentration (nM)		24h		48h	
ABT-199	HHT	Fa	CI	Fa	CI
1.25	2	0.352	5.011	0.446	0.253
2.5	4	0.553	0.322	0.528	0.321
5	8	0.582	0.383	0.660	0.620
10	16	0.642	0.120	0.573	0.071
20	32	0.703	0.061	0.694	0.023
MOLM13					
Concentration (nM)		24h		48h	
ABT-199	HHT	Fa	CI	Fa	CI
1.25	1	0.121	0.2705	0.110	0.4823
2.5	2	0.210	0.1653	0.197	0.2216
5	4	0.256	0.2125	0.312	0.1411
10	8	0.374	0.1711	0.485	0.0845
20	16	0.442	0.2184	0.687	0.0467
OCL-AML2					
Concentration (nM)		24h		48h	
ABT-199	HHT	Fa	CI	Fa	CI
10	2	0.135	0.097	0.089	0.584
20	4	0.188	0.061	0.121	0.441
40	8	0.207	0.083	0.189	0.205
80	16	0.413	0.011	0.488	0.009
160	32	0.541	0.005	0.566	0.008
320	64	0.641	0.003	0.766	0.001
OCL-AML3					
Concentration (nM)		24h		48h	
ABT-199	HHT	Fa	CI	Fa	CI
10	2	0.039	0.341	0.054	0.489
20	4	0.033	0.992	0.064	0.753
40	8	0.075	0.312	0.168	0.318
80	16	0.522	0.003	0.648	0.028
160	32	0.688	0.002	0.838	0.014

CI, Combination index.

Fa, Fractional inhibition.

**Table 3:** The characteristics of 5 cases diagnosed with *de novo* or refractory/relapsed acute myeloid leukemia.

Patient	Sex	Age	Cell count(10^9)			LDH(U/L)	Karyo-type	Molecular features
No.		(yr)	WBC	HB	PLT			
1	F	44	28.1X10^9/L	66.0g/L	73.0X10^9/L	256/L	46, XY	No
2	M	29	54.15	77.0	43.0	255	46, XY, t(11;19)	No
3	M	27	20.50	69.0	63	691	46, t(8;21)	AML-ETO
4	M	62	44.43	43.0	204	226	ND	No
5	M	65	49.29	162	43.0	432	46, XY	No

### Effects of ABT-199 Combined With HHT on Colony Formation

**Figure 3:**
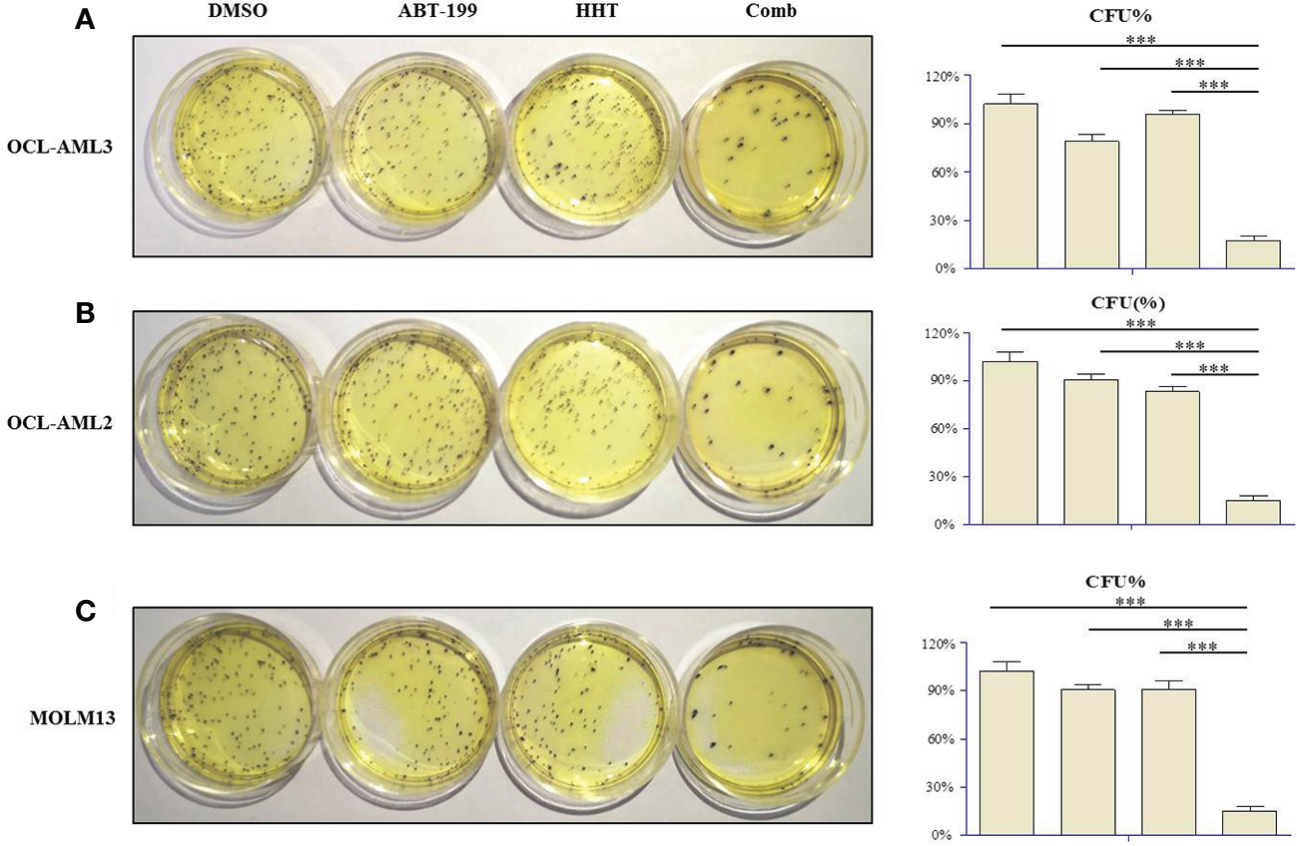
The numbers of colony-forming units (CFU) produced by OCL-AML2, OCL-AML3, and MOLM13 cells exposed to ABT-199 alone (80 nM) or in combination with HHT (16 nM) in a methylcellulose culture system for 24 h **(A–C)**. The percentage of CFU was determined by counting colonies (≥50 cells). Data are presented as the mean ± S.D. @ of three independent experiments. (*P < 0.05, **P < 0.01, and ***P < 0.001).

Clonogenicity assays were carried out to investigate whether ABT-199 combined with HHT affects the clonogenic capacity of AML cells. Therefore, OCL-AML2, OCL-AML3, and MOLM-13 cells were treated with the indicated concentrations of ABT-199 or HHT alone or in combination for 24 h. Neither ABT-199 (80 nM) nor HHT (16 nM) alone diminished the colony formation abilities of the OCL-AML2, OCL-AML3, and MOLM-13 cells **(** **Figures 3A–C** **)**. The colony formation of MV4-11 was listed in the **Supplemental Figure 5**. However, when the combination of ABT-199 and HHT was given, the colony-forming units decreased remarkably (P<0.001 *vs.* control, ABT-199 alone, or HHT alone).

### Combination Therapy With ABT-19 and HHT Was More Active Than Either Monotherapy in a Xenograft Mouse Model

**Figure 4:**
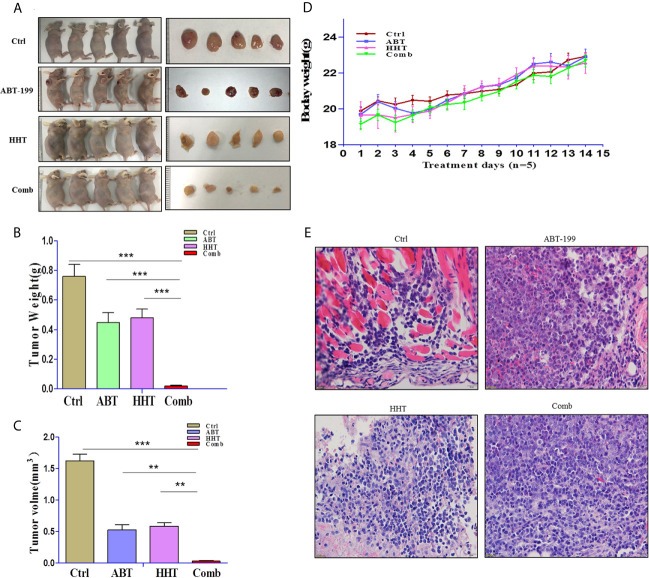
The antitumor potency of ABT-199 combined with HHT was evaluated in OCL-AML3 cell line-derived mouse xenograft models (n = 5 per group). Compared with each agent alone, the combination of ABT-199 with HHT significantly decreased the tumor burden, including tumor size and weight **(A–C)**. Mouse body weight was measured every other day. **(D)** Bone marrow from mice was embedded in paraffin and stained with H&E. Scale bar, 25 μm. Data represent the mean ± S.D. *P < 0.05 *vs* the cotreatment groups **(E)**. (*P < 0.05, **P < 0.01, and ***P < 0.001).

To validate that the joint effects of ABT-199 and HHT measured *in vitro* translate into a difference in tumor responsiveness *in vivo*, we established a xenograft mouse model by subcutaneous injection of OCL-AML3 cells. As we expected, the results were consistent with those of the *in vitro* experiments. Mice treated with ABT-199 combined with HHT showed a remarkably reduced tumor burden **(** **Figures 4A–C** **)**. Moreover, the combined treatment group showed little lethal toxicity **(** **Figure 4D** **)**. Consistently, histopathological analysis revealed a remarkable reduction in leukemia cell infiltration into tumor tissue in the combination group **(** **Figure 4E** **)**. In brief, the combination regimen of ABT-199 and HHT was more effective than the corresponding single-agent treatments in inhibiting AML growth and progression.

### ABT-199 Combined With HHT Modulated MCL-1 Phosphorylation by Inhibiting p-ERK and Activating BAX

**Figure 5:**
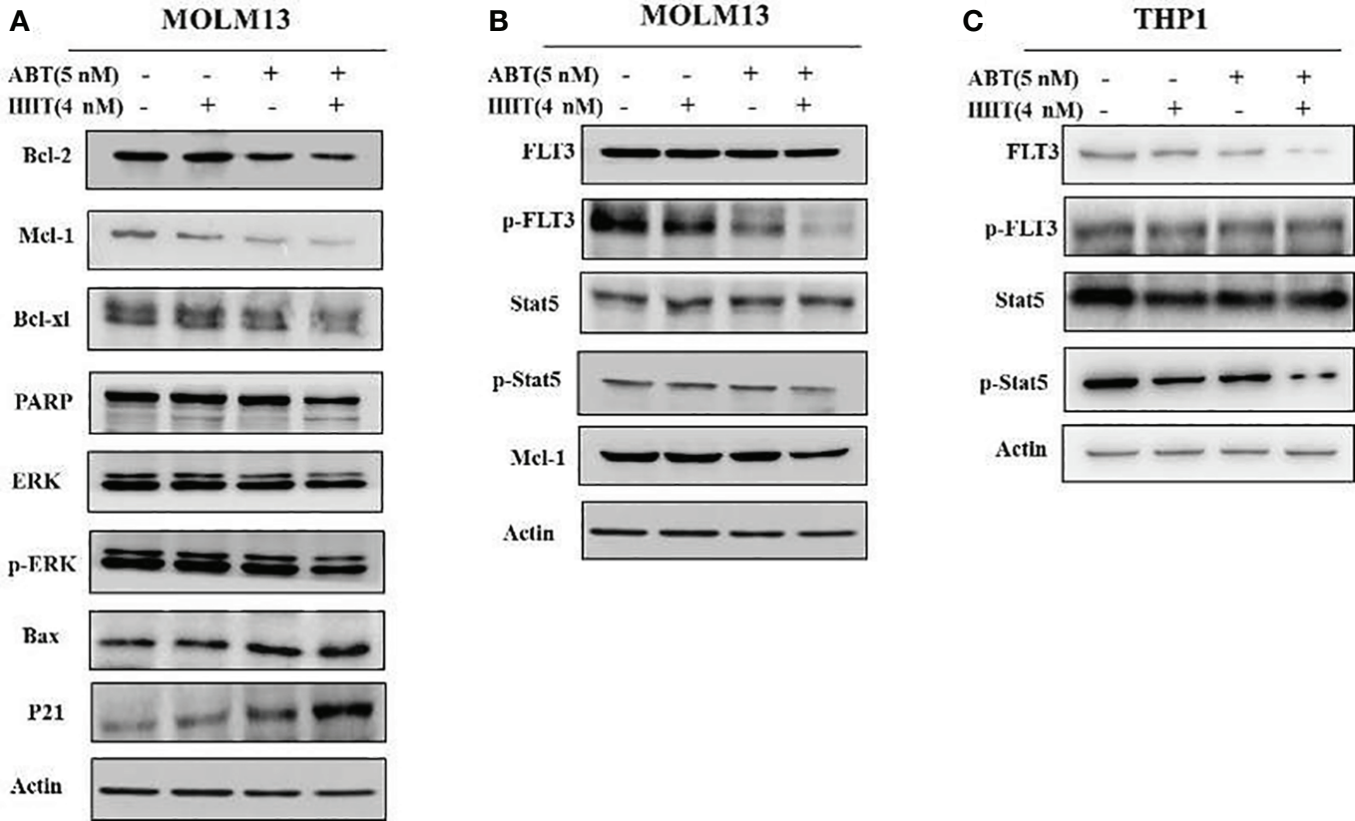
In the MOLM-13 cell lines, compared with each agent alone, ABT-199 combined with HHT downregulated MCL-1, p-ERK expression **(A)**. Strikingly, our results also showed that cotreatment with ABT-199 and HHT inhibited the FLT3/Stat5/MCL-1 signaling cascade, and the protein levels of p-FLT3, p-Stat5, and MCL-1 were determined by Western blotting **(B)**. The total and phosphorylation protein of FLT3 and STAT in a FLT3 ITD-negative cell line were shown by Western blot **(C)**.

To better understand the underlying mechanism of the reductions in BCL-2 and MCL-1, Western blotting was performed, and the results showed that the reductions in the MCL-1 protein levels in AML cell lines treated with HHT might be mediated through proteasome degradation. ABT-199 has been reported to associate with BAX. We found that ABT-199 combined with HHT reduced MCL-1 level but increased BAX level. In addition, compared with ABT-199 or HHT alone, combination treatment produced more p-ERK degradation. The results showed that ABT-199 combined with HHT modulated MCL-1 phosphorylation by inhibiting p-ERK and activating BAX **(** **Figure 5A** **)**.

### ABT-199 Combined With HHT Downregulated the FLT3/Stat5/MCL-1 Signaling Cascade

To investigate the cytotoxicity mechanism of ABT-199 combined with HHT in AML cells, potential signals were further analyzed by Western blot analysis. As we have mentioned above ABT-199 combined with HHT are sensitive to FLT3 mutant cell lines so ABT-199 combined with HHT markedly reduced the phosphorylation of FLT3 in the MOLM13 cell line **(** **Figure 5B** **)**. However, the FLT3 downstream signaling molecules total Stat5 did not significantly change. Our results showed that ABT-199 combined with HHT yielded substantial reductions in the levels of p-FLT3 and p-Stat5 in MOLM13 cells. The total and phosphorylation protein of FLT3 and STAT in a FLT3 ITD-negative cell line were shown in **Figure 5C**.

## Discussion

Chemotherapy is currently one of the main approaches for AML therapy. However, most targeted agents concentrate on upstream nodes in cancer signaling pathways. Drug resistance and severe side effects as well as relatively low overall survival limit the use of traditional chemotherapeutic drugs (14, 15). Drug resistance is usually inevitable, particularly for monotherapies utilizing targeted compounds, due to complex cancer signaling pathways (16, 17). Studies have shown that FLT3-ITD MR is related to complete remission (CR) and overall survival (OS) in AML patients. Furthermore, FLT3-ITD MR may act as an independent prognostic factor for OS in non-M3 AML patients. Classifying risk grades based on FLT3-ITD MR is crucial for individualized treatment and prognostic evaluation. Concordantly, monotherapy with ABT-199 has a finite efficacy because of the absence of triggering of BH3-only protein expression and compensatory upregulation of MCL-1 expression (18, 19). Combining two agents is an effective way to reduce the dose used at this stage. An ideal combination of two drugs would be able to enhance proapoptotic effects, such as inducing the expression of the Bax protein. According to our research, we found that ABT-199 combined with HHT exerted superior synergistic lethality in AML cell lines.

HHT, a natural alkaloid derived from Cephalotaxus, is widely applied for AML therapy in China (12, 20–23). Recently, some researchers launched a national, multicenter, randomized, double-blinded, prospective phase III clinical trial to study the effect of an HHT-based induction regimen on *de novo* AML patients. The results showed that the HHT-based regimen achieved a relatively high completion rate and prolonged overall survival (24). Additionally, HHT plays an important role in the treatment of chronic myeloid leukemia (CML). In 2010, FAD evaluated the use of HHT in relapsed/refractory CML. Clinical research on the mechanisms of action of HHT has found different mechanisms, including binding with the small subunit of the ribosome and interfering with the process of translation to inhibit protein synthesis (25). However, adverse events have been observed to be similar in all groups studied (26). Tumor recurrence and drug resistance are associated with high expression of anti-apoptotic proteins, such as MCL-1, that have been increasingly recognized as important targets in cancer therapy (8). In addition, because the MCL-1 protein has a short half-life, it can be easily cleaved by activated caspases during apoptotic cell death (27). Numerous experiments have shown that co-treatment with other antitumor therapeutics can reduce the level of MCL-1. It was recently reported that the anti-apoptotic activity of MCL-1 is necessary for the development and sustained growth of AML (28). If a drug can effectively inhibit MCL-1, then it should have some effects on AML.

BCL-2 was initially found in lymphoid cancer cells. A large number of studies on BCL-2 have been conducted in lymphoid cells, in which BCL-2 is highly expressed (29, 30). In our research, we found that selective, on-target BCL-2 inhibition was a superior method for the clinical treatment of AML (31). It should be noted that even AML myeloblasts that are not sensitive to conventional chemotherapy appear to be quite sensitive to BCL-2 inhibitors (32). Thus, the BCL-2 inhibitor ABT-199, an effective chemotherapeutic agent, has been used in the clinic. In our research, we found that Bax expression was dramatically upregulated, whereas BCL-2 and MCL-1 levels were downregulated in ABT-199 plus HHT combination-treated cells, leading to a dramatic increase in the cleavage of caspase-3, which may be one of the ways that HHT enhances the promotive effect of ABT-199 on apoptosis in AML cell lines.

Our results found that ABT-199 combined with HHT could inhibit those with FLT3-ITD mutant of AML cell proliferation by inducing apoptosis in a dose- and time-dependent manner. Furthermore, the possible mechanism revealed inhibition of the antiapoptotic proteins BCL-2 and MCL-1 and activation of caspase family members, such as caspase-3 and caspase-9. Cell cycle blockade inhibits DNA synthesis, thereby inhibiting cell proliferation and promoting antileukemic effects (33). The critical role of PI3K signaling in the progression of numerous tumors, including leukemia, has been well reported (34, 35). Strikingly, we revealed that ABT-199 combined with HHT could effectively inhibit the expression of p-FLT3 and its downstream signaling proteins, p-Stat5 and MCL-1, inducing apoptosis in AML cell lines.

Interestingly, according to the IC50 value, we observed a phenomenon in which MV4-11 and MOLM13 cells carried the FLT3-ITD mutation and exhibited increased sensitivity to ABT-199, especially when combined with HHT. It has been reported that HHT affects the FLT3-STAT5 signaling pathway (36). Our research showed that HHT, especially in combination with ABT-199, had a significant effect on the FLT3 signaling pathway by downregulating the phosphorylation of FLT3 and STAT5. In this study, the combination of ABT-199 and HHT exerted promising antileukemic effects at the lower tested doses. The low but effective doses of ABT-199 combined with HHT likely observed *in vitro* may produce tolerance advantages *in vivo*. Overall, our study provided a rationale for a novel combination approach to cure AML. Besides, our research indicated that HHT could strengthen the antileukemic effect of ABT-199 *in vitro* and *in vivo*. In addition, we also discussed the potential mechanisms of the two drugs. Therefore, our findings provide a strong rationale for a phase I/II clinical trial with ABT-199 and HHT combination treatment of AML patients.

## Conclusion

Taken together, our research results show that ABT-199 combined with HHT exerts antileukemic activity *in vitro* and *in vivo*, likely through inhibiting the expression of BCL-2 and MCL-1, as well as the FLT3-STAT5 signaling pathway, and provide potential benefits and a clinical application approach for ABT-199 and HHT in AML patients.

## Data Availability Statement

The original contributions presented in the study are included in the article/**Supplementary Material**. Further inquiries can be directed to the corresponding author.

## Ethics Statement

The animal study was reviewed and approved by Department of Hematology, The First Affiliated Hospital, College of Medicine, Zhejiang University.

## Author Contributions

YS and JY performed the experiments and analysis of data. YY and HS collected primary AML samples and interpret data and wrote the manuscript. XY and WX contributed to study design, data analysis, and interpretation and manuscript revision. All authors contributed to the article and approved the submitted version.

## Funding

This work was supported in part by the Research Plan of the National Natural Science Foundation of China (No. 81372256).

## Conflict of Interest

The authors declare that the research was conducted in the absence of any commercial or financial relationships that could be construed as a potential conflict of interest.
