# Supplementary material for: The Basic Research of the Combinatorial Therapy of ABT-199 and Homoharringtonine on Acute Myeloid Leukemia
Source: Front Oncol. 2021 Jul 14;11:692497. doi: 10.3389/fonc.2021.692497 (PMC8317985; doi:10.3389/fonc.2021.692497)
Supplement: Supplementary file 1 [file DataSheet_1.zip › Supplementary Figure 1.DOCX]

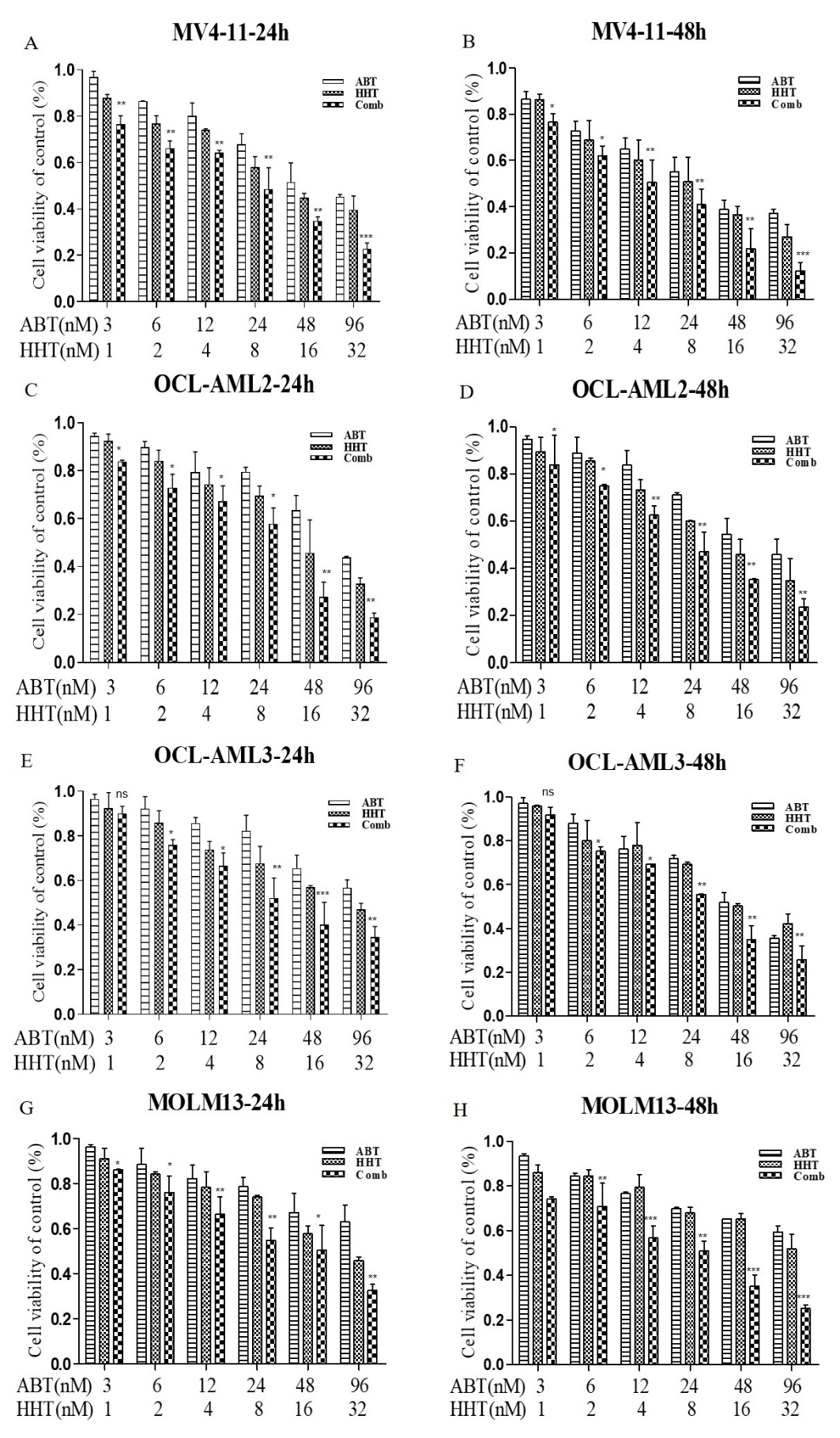


**Supplemental Figure 1**

AML cell lines (OCL-AML2, OCL-AML3, MOLM13, and MV4-11) were treated with various doses of ABT-199 or HHT alone or in combination for 24 h or 48 h in 3:1 concentration (A-H). The percent viability is normalized to the percent viability of the DMSO-treated control. Values are expressed as the mean ± S.D. of three independent experiments.
