# Supplementary material for: The Basic Research of the Combinatorial Therapy of ABT-199 and Homoharringtonine on Acute Myeloid Leukemia
Source: Front Oncol. 2021 Jul 14;11:692497. doi: 10.3389/fonc.2021.692497 (PMC8317985; doi:10.3389/fonc.2021.692497)
Supplement: Supplementary file 1 [file DataSheet_1.zip › Supplementary Figure 3.DOCX]

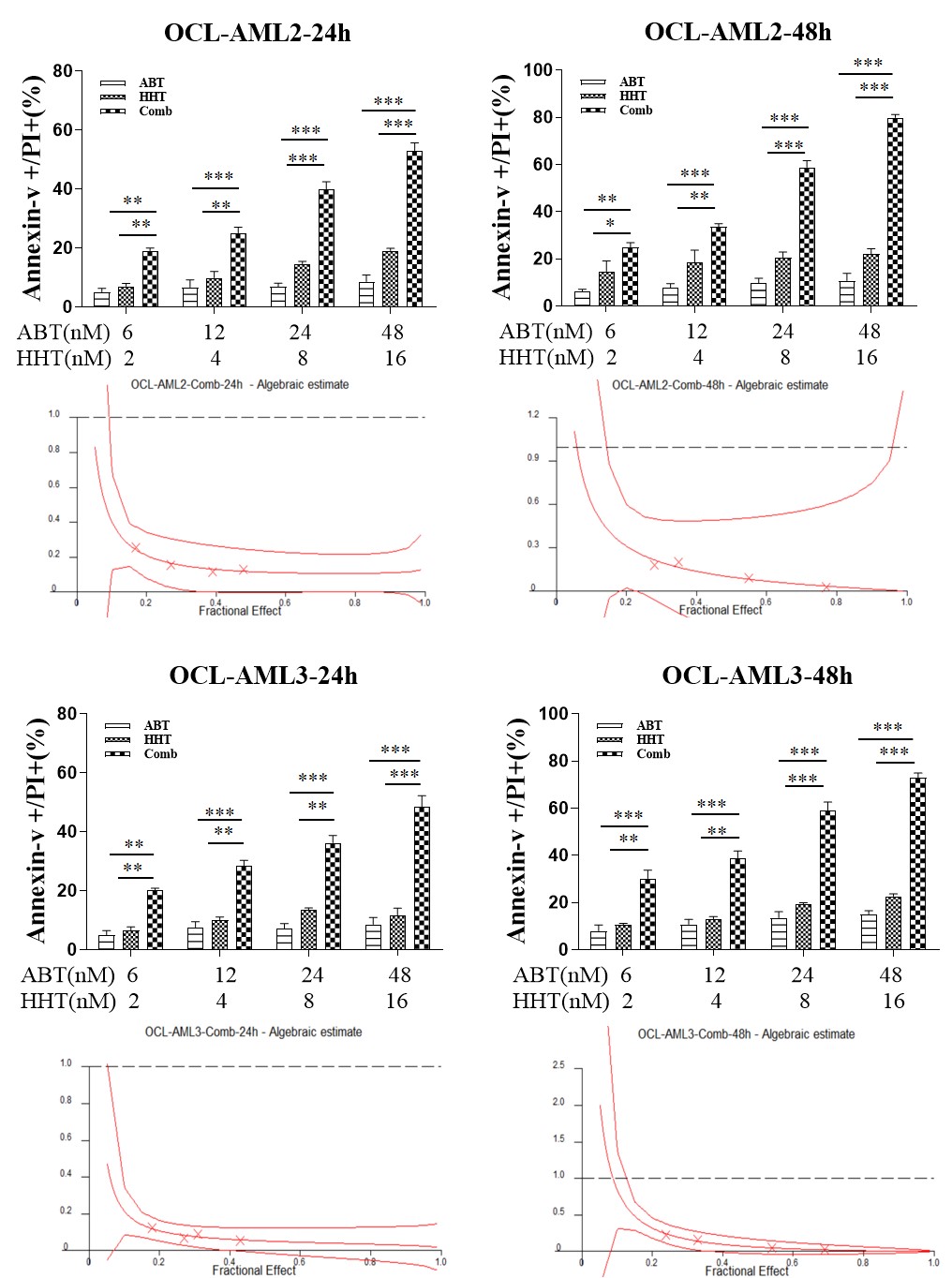


**Supplemental Figure 3**

The percentage of apoptotic cells was examined with a NovoCyte flow cytometer. ABT-199 combined with HHT resulted in significant increases in the apoptosis rate in AML cell lines in 3:1 concentration.
