# Supplementary material for: The Basic Research of the Combinatorial Therapy of ABT-199 and Homoharringtonine on Acute Myeloid Leukemia
Source: Front Oncol. 2021 Jul 14;11:692497. doi: 10.3389/fonc.2021.692497 (PMC8317985; doi:10.3389/fonc.2021.692497)
Supplement: Supplementary file 1 [file DataSheet_1.zip › Supplementary Figure 5.DOCX]

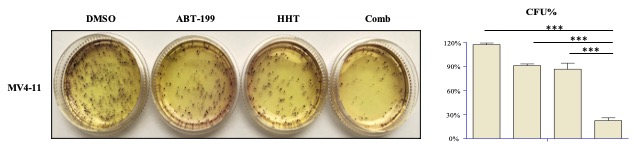


**Supplemental Figure 5**

The numbers of colony-forming units (CFU) produced by MV4-11 cells exposed to ABT-199 alone (80 nM) or in combination with HHT (16 nM) in a methylcellulose culture system for 24 h. The percentage of CFU was determined by counting colonies (≥50 cells). Data are presented as the mean ± S.D. of three independent experiments.
