# Supplementary figures and images for: The Basic Research of the Combinatorial Therapy of ABT-199 and Homoharringtonine on Acute Myeloid Leukemia
Source: Front Oncol. 2021 Jul 14;11:692497. doi: 10.3389/fonc.2021.692497 (PMC8317985; doi:10.3389/fonc.2021.692497)

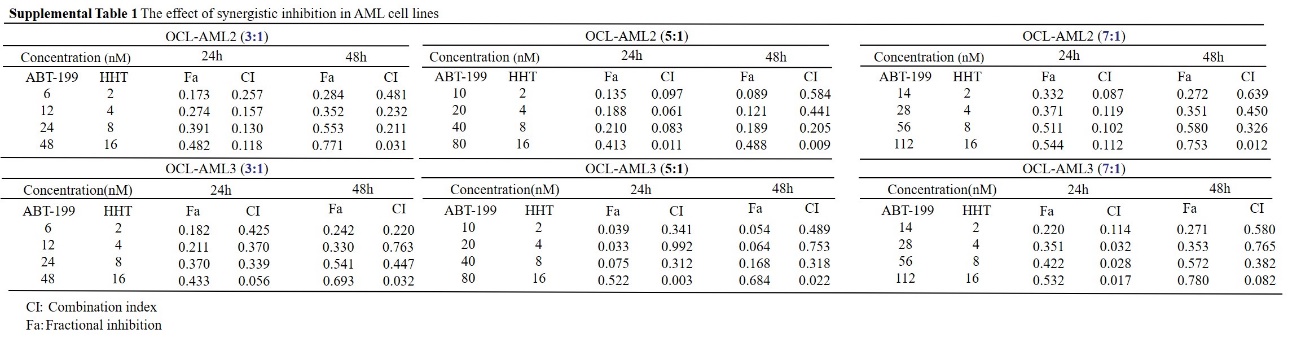


**Supplemental Table 1**

The effect of synergistic inhibition of ABT-199 and HHT in 3:1, 5:1, and 7:1 in AML cell lines.

Supplement: Supplementary file 1 [file DataSheet_1.zip › Supplementary Table 1.DOCX]
